# Integrating Structural and Functional Interhemispheric Brain Connectivity of Gait Freezing in Parkinson's Disease

**DOI:** 10.3389/fneur.2021.609866

**Published:** 2021-04-15

**Authors:** Chaoyang Jin, Shouliang Qi, Yueyang Teng, Chen Li, Yudong Yao, Xiuhang Ruan, Xinhua Wei

**Affiliations:** ^1^College of Medicine and Biological Information Engineering, Northeastern University, Shenyang, China; ^2^Key Laboratory of Intelligent Computing in Medical Image, Ministry of Education, Northeastern University, Shenyang, China; ^3^Department of Electrical and Computer Engineering, Stevens Institute of Technology, Hoboken, NJ, United States; ^4^Department of Radiology, Guangzhou First People's Hospital, School of Medicine, South China University of Technology, Guangzhou, China

**Keywords:** Parkinson's disease, freezing of gait, diffusion tensor imaging, resting state fMRI, voxel-mirrored homotopic connectivity

## Abstract

Freezing of gait (FOG) has devastating consequences for patients with Parkinson's disease (PD), but the underlying pathophysiological mechanism is unclear. This was investigated in the present study by integrated structural and functional connectivity analyses of PD patients with or without FOG (PD FOG+ and PD FOG–, respectively) and healthy control (HC) subjects. We performed resting-state functional magnetic resonance imaging (fMRI) and diffusion tensor imaging of 24 PD FOG+ patients, 37 PD FOG– patients, and 24 HCs. Tract-based spatial statistics was applied to identify white matter (WM) abnormalities across the whole brain. Fractional anisotropy (FA) and mean diffusivity (MD) of abnormal WM areas were compared among groups, and correlations between these parameters and clinical severity as determined by FOG Questionnaire (FOGQ) score were analyzed. Voxel-mirrored homotopic connectivity (VMHC) was calculated to identify brain regions with abnormal interhemispheric connectivity. Structural and functional measures were integrated by calculating correlations between VMHC and FOGQ score and between FA, MD, and VMHC. The results showed that PD FOG+ and PD FOG– patients had decreased FA in the corpus callosum (CC), cingulum (hippocampus), and superior longitudinal fasciculus and increased MD in the CC, internal capsule, corona radiata, superior longitudinal fasciculus, and thalamus. PD FOG+ patients had more WM abnormalities than PD FOG– patients. FA and MD differed significantly among the splenium, body, and genu of the CC in all three groups (*P* < 0.05). The decreased FA in the CC was positively correlated with FOGQ score. PD FOG+ patients showed decreased VMHC in the post-central gyrus (PCG), pre-central gyrus, and parietal inferior margin. In PD FOG+ patients, VMHC in the PCG was negatively correlated with FOGQ score but positively correlated with FA in CC. Thus, FOG is associated with impaired interhemispheric brain connectivity measured by FA, MD, and VMHC, which are related to clinical FOG severity. These results demonstrate that integrating structural and functional MRI data can provide new insight into the pathophysiological mechanism of FOG in PD.

## Introduction

Freezing of gait (FOG), which is among the most serious symptoms of Parkinson's disease (PD) (1), is defined as the inability to achieve an effective gait when walking (2). PD with FOG (PD FOG+) is characterized by severe and sudden gait disorder, with patients describing a feeling that their feet are glued to the floor. The duration of FOG is usually a few seconds but occasionally lasts for dozens of seconds or more. Unlike other cardinal symptoms, FOG is difficult to manage with dopaminergic drugs or deep brain stimulation (3).

FOG is considered a mysterious clinical phenomenon, and the underlying pathophysiological mechanism is unclear (1, 2, 4). One explanation that has been proposed is that the difficulty of performing movements autonomously results in an increased reliance on attention to execute movements (5). It has also been suggested that executive dysfunction prevents PD patients from performing an action when it is required (6). Impaired control of rhythmicity, bilateral coordination, and gait asymmetry are also important aspects of freezing (7).

Brain white matter (WM) abnormalities in PD FOG+ rarely overlap. Altered WM connectivity has been observed in the bilateral pedunculopontine nucleus and superior premotor cortex and left supplementary motor area (8). One study found no difference in the tract projecting from the supplementary motor region to subcortical regions between PD FOG+ and PD without FOG (PD FOG–) (9). However, damage to multiple cortical areas involved in high-level gait control along with WM disruption in motor, cognitive, and limbic structures may constitute the anatomic correlates of FOG (10).

Voxel-mirrored homotopic connectivity (VMHC) analysis based on resting-state functional magnetic resonance imaging (fMRI) data has been proposed as a means of measuring the synchronization of spontaneous neural activity [functional connectivity (FC)] between interhemispheric regions (11). Specifically, Pearson's correlation coefficient between the time series of the low-frequency (0.01–0.08 Hz) blood oxygen level-dependent signal of each voxel and that of its spatially symmetric counterpart in the contralateral hemisphere is calculated, and the VMHC value is calculated by Fisher's Z transformation and weighting by voxel-wise gray matter probability. Further group-level analysis is performed to identify clusters or brain regions with significantly different VMHC (12, 13). A decreased VMHC has been reported in PD in brain regions related to information processing, sensory integration, and motor coordination (11) including the putamen and cortex (14). Moreover, PD FOG+ patients were shown to have lower VMHC in the inferior parietal lobe than PD FOG– patients (15).

Few studies have investigated the etiology of FOG in PD by integrating structural and functional brain connectivity data because of the difficulty of data acquisition. Tract-based spatial statistics (TBSS) and independent component analysis have been used to investigate abnormalities in structural connectivity and FC, respectively (16). However, no WM differences have been observed between PD FOG+ and PD FOG– patients (17). In another study, no structural abnormalities were found in PD FOG+ patients by TBSS and FC analysis (2).

In the present study, we used TBSS to examine structural defects in the brain of PD patients with or without FOG. Fractional anisotropy (FA) and mean diffusivity (MD) in the splenium, body, and genu of the corpus callosum (CC) were compared between PD FOG+ and PD FOG– patients and healthy control (HC) subjects, and the correlations between these parameters and clinical severity as reflected by FOG Questionnaire (FOGQ) score (18) were analyzed. We also used VMHC to identify regions with abnormal interhemispheric FC. After evaluating the correlation between VMHC and FOGQ score, correlations between VMHC, FA, and MD were determined to integrate structural and functional indices.

## Methods

### Participants

A total of 61 right-handed PD patients were recruited for this study. All patients met the Parkinson's diagnostic criteria of the UK Brain Bank. The exclusion criteria were as follows: (1) previous history of cerebrovascular disease, brain injury, or other neurodegenerative disease; (2) PD co-occurring with diseases that seriously affect gait such as eye disease, osteoarthritis, and neuromuscular disease; (3) severe cognitive impairment or dementia [Mini-mental State Examination (MMSE) score <24]; and (4) major diseases or contraindications for MRI.

Patients who scored >1 point on the third item of the FOGQ or who were determined to have FOG by more than two experienced neurologists in a series of motion tests were considered as PD FOG+. Of the 61 PD patients, 24 were PD FOG+ and 37 were PD FOG–. Additionally, 24 right-handed HCs who were matched to the patients in terms of age, sex, and education level were recruited. The study was approved by the ethics committee of Guangzhou First People's Hospital and was carried out in accordance with the 1964 Helsinki Declaration and its later amendments or comparable ethical standards. All subjects were informed of and understood the study protocol and signed the informed consent form prior to participation in the study.

### Clinical Assessment

All patients were assessed in terms of motor, visuospatial, cognitive, executive, and attention functions. The Unified Parkinson's Disease Rating Scale (UPDRS-III) (19) and Hoehn and Yahr (H&Y) scale were used to assess the severity of motor symptoms (20). The Timed Up and Go (TUG) test was used to evaluate patients' mobility (21). The FOGQ was used to assess the severity of freezing. MMSE, Montreal Cognitive Assessment (MoCA), and Frontal Assessment Battery (FAB) (22) were used to evaluate cognitive function; and the mental state of the participants was assessed with the Hamilton Depression Rating Scale (HDRS) and Hamilton Anxiety Rating Scale (HARS).

Age and education level were compared among the three groups by one-way analysis of variance, and the chi-squared test was used to compare sex ratios. The two-sample *t*-test was used for comparisons of disease duration and scores for UPDRS-III, H&Y scale, FOGQ, MMSE, MOCA, FAB, TUG, HDRS, and HARS.

### MRI Data Acquisition

MRI images of PD patients were collected in the “off” state (i.e., patients had stopped taking anti-Parkinson drugs for at least 12 h) to eliminate the effects of drugs on neural activity. MRI scans were performed with a 3.0-T MAGNETOM Verio whole-body MRI system (Siemens, Munich, Germany) equipped with eight-channel phase-array head coils. Tight foam padding was used to minimize head movement, and earplugs were used to reduce noise. Subjects were instructed to remain motionless, close their eyes and remain awake, and avoid thinking about anything.

Three-dimensional (3D) T1-weighted images were acquired with a 3D magnetization-prepared rapid gradient echo sequence [repetition time (TR) = 1,900 ms, echo time (TE) = 102 ms, flip angle = 9°, thickness = 1.0 mm, slices = 160, field of view (FOV) = 250 × 250 mm^2^, matrix = 256 × 256, and voxel size = 1.0 × 1.0 × 1.0 mm^3^]. Resting-state fMRI images are collected with an echo-planar imaging (EPI) sequence (TR = 2,000 ms, TE = 21 ms, flip angle = 78°, FOV = 256 × 256 mm^2^, matrix = 64 × 64, slices = 220, thickness = 4.0 mm, and voxel size = 3.5 × 3.5 × 4.0 mm^3^). Diffusion tensor imaging (DTI) was performed with a spin EPI sequence (TR = 8,700 ms, TE = 102 ms, FOV = 230 × 230 mm^2^, voxel size = 2.5 × 2.5 × 2.5 mm^3^, matrix = 92 × 92, thickness = 2.5 mm, and slice gap = 0 mm). Diffusion gradients were applied in 30 non-collinear directions with a b factor of 2,000 s/mm^2^ after acquisition with b = 0 s/mm^2^.

### Overview of the Study Protocol

**Figure 1:**
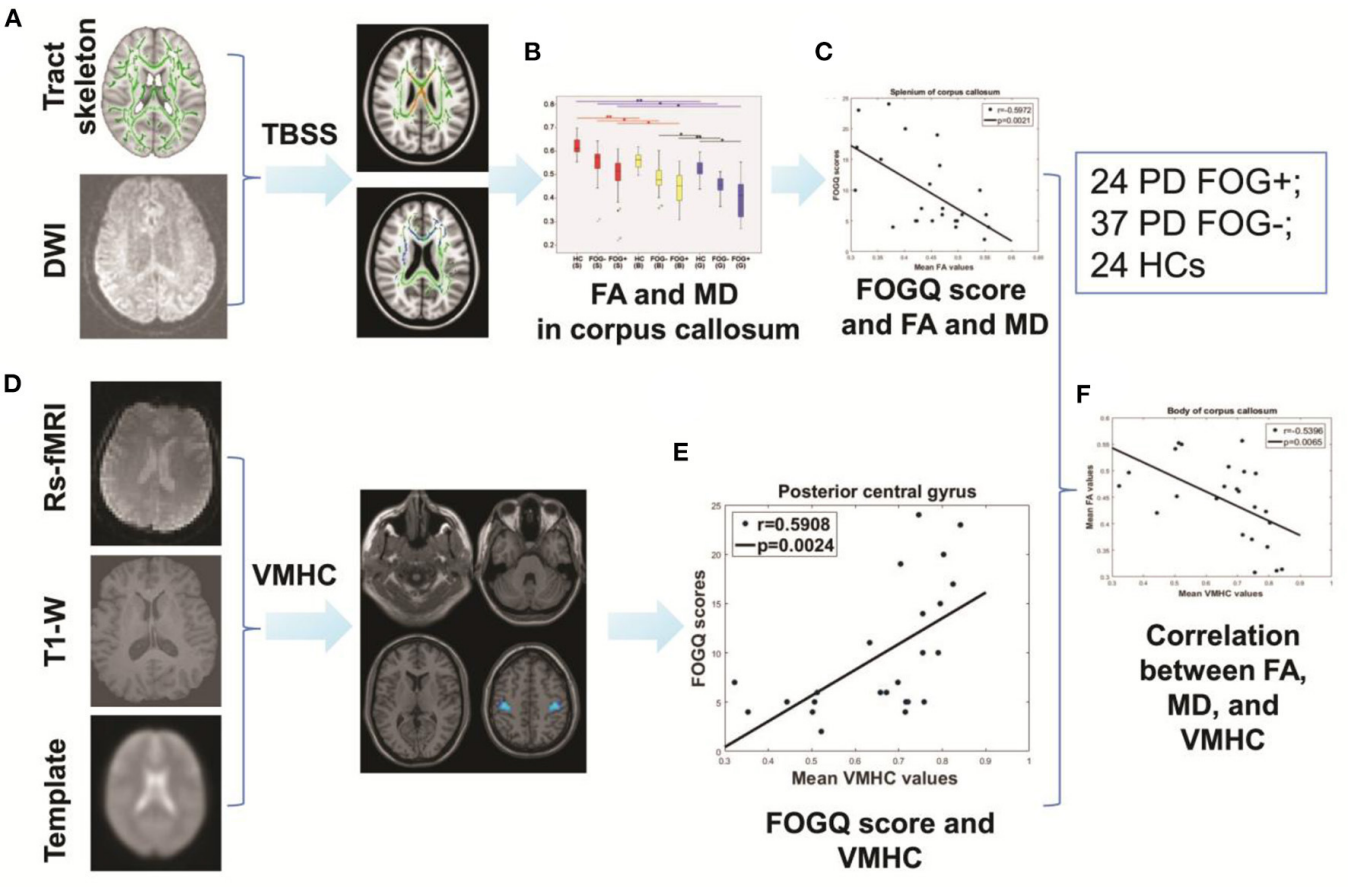
Study protocol. **(A)** TBSS. **(B)** Intergroup comparisons of FA and MD in the CC. **(C)** Correlation between FA, MD, and FOGQ scores. **(D)** Analysis of VMHC. **(E)** Correlation between FOGQ scores and VMHC. **(F)** Correlation between FA, MD, and VMHC.

The study consisted of the following six steps (Figure 1): (1) Image processing was carried out for resting-state fMRI and DTI data; (2) TBSS were used to identify WM abnormalities throughout the whole brain; (3) the two-sample *t*-test was used to evaluate differences in FA and MD in the splenium, corpus, and genu of the CC across the three groups; (4) a correlation analysis was performed between FA, MD, and FOGQ scores in the CC; (5) a VMHC analysis was performed to explore homotopic connectivity; and (6) correlations between FA, MD, and VMHC were analyzed.

### TBSS and Analysis of FA, MD, and FOGQ Score

DTI data were preprocessed and analyzed using the Pipeline for Analyzing Brain Diffusion Images toolkit (http://www.nitrc.org/projects/panda). FA and MD maps were obtained after standard preprocessing including eddy current correction and brain extraction (23).

The TBSS procedure consisted of four steps: (1) The non-linear registration method was used to register each FA image to the target template; (2) all registered FA images were used to generate the average FA template, extract its skeleton, and remove smaller edge fiber bundles according to a threshold value of 0.2; (3) FA in each standard space was mapped to the FA skeleton map; and (4) the Tbss-no_FA tool was for TBSS analysis of MD images. Family-wise error correction was applied to the statistical results of TBSS. The threshold-free cluster enhancement correction method was used for the correction of multiple comparisons. The number of permutations was set to 5,000, and differences with *P* < 0.05 were considered statistically significant.

The Johns Hopkins University ICBM-DTI-81 WM atlas was used to identify areas of WM impairment. FA and MD in these areas were compared among groups with the two-sample *t*-test, and their correlations with FOGQ score were analyzed. False discovery rate (FDR) correction was applied to multiple comparisons of FA and MD.

### Preprocessing of fMRI Data

Data processing assistant for resting-state fMRI was used to preprocess the fMRI data (24). The first 10 time points were removed. The head movement standard was set to 2 mm (translation) or 2° (rotation). The brain was segmented into gray matter, WM, and cerebrospinal fluid (CSF). After head movement correction, the images were standardized and resampled to 3 × 3 × 3 mm^3^. Linear regression was used to remove the influence of six head movement parameters and WM and CSF signals (25). A Gaussian kernel with 6-mm full width at half maximum was used for spatial smoothing. The data were detrended and filtered at low frequency (0.01–0.08 Hz).

### VMHC and FOGQ Score and Integration of FA, MD, and VMHC

VMHC was calculated using Resting-State fMRI Data Analysis Toolkit plus software package [(26); http://www.restfmri.net]. After obtaining the time series of each voxel, homotopic FC was calculated as the Pearson correlation coefficient between each voxel and the contralateral mirror voxel. The coefficients were then converted into T values with Fisher's Z transformation for normalization; these values represented VMHC (27).

In the VMHC map, the mean value was standardized to reduce differences between subjects. Analysis of covariance was carried out to identify brain regions with significant differences among the three groups. Using the resultant regions as masks, the two-sample *t*-test and multiple comparison correction of the Gaussian random field (voxel *P* < 0.001, cluster *P* < 0.05) were performed to evaluate differences in VMHC between groups.

To clarify the relationship between interhemispheric connectivity and FOG severity, the correlation between VMHC in regions with WM abnormalities and FOGQ score was analyzed. To explore the relationship between CC abnormalities and interhemispheric connectivity of the post-central gyrus (PCG), correlations between MD and FA of the CC and VMHC of the PCG were determined.

## Results

### Demographic and Clinical Characteristics of the Study Population

Demographic and clinical characteristics of the participants are shown in Table 1. There were no significant differences between PD FOG+ and PD FOG– groups in terms of age (*P* = 0.298), education level (*P* = 0.36), sex ratio (*P* = 0.452), disease duration (*P* = 0.062), UPDRS-III score (*P* = 0.19), and H&Y scale score (*P* = 0.16). As expected, FOGQ, FAB, and TUG scores differed between PD FOG+ and PD FOG– patients (*P* < 0.001), but there were no differences in MMSE (*P* = 0.46), MoCA (*P* = 0.353), HDRS (*P* = 0.34), or HARS (*P* = 0.76) scores between the two groups.

**Table 1:** Demographic and clinical characteristics of participants.

**Parameter**	**HC (*n* = 24)**	**PD FOG+ (*n* = 24)**	**PD FOG– (*n* = 37)**	***P*-value**
Age, years	62.5 ± 3.8	65.5 ± 6.1	64.1 ± 8.2	0.298^a^
Education, years	10.98 ± 2.34	9.35 ± 3.42	10.65 ± 4.25	0.36^a^
Sex, female/male	15/9	11/13	18/19	0.452^b^
Disease duration, years	NA	6.00 ± 5.25	3.01 ± 3.21	0.062^c^
UPDRS-III	NA	22.4 ± 6.72	21.34 ± 10.30	0.19^c^
H&Y scale	NA	2.49 ± 0.51	2.07 ± 0.49	0.16^c^
FOGQ	NA	9.25 ± 5.87	1.54 ± 1.67	<0.001^c^*
MMSE	NA	25.27 ± 4.01	25.71 ± 4.25	0.46^c^
MoCA	NA	21.08 ± 4.77	21.89 ± 5.66	0.353^c^
FAB	NA	13.8 ± 2.6	15.7 ± 1.5	<0.001^c^*
TUG	NA	12.5 ± 1.6	1.9 ± 0.7	<0.001^c^*
HDRS	NA	7.82 ± 6.35	9.67 ± 6.23	0.34^c^
HARS	NA	11.32 ± 6.74	10.56 ± 7.56	0.76^c^

*Data are shown as mean ± standard deviation*.

* *P < 0.05*.

a *One-way analysis of variance*.

b *Chi-squared test*.

c *Two-sample t-test*.

*FAB, Frontal Assessment Battery; FOGQ, Freezing of Gait Questionnaire; H&Y, Hoehn and Yahr; HARS, Hamilton Anxiety Rating Scale; HDRS, Hamilton Depression Rating Scale; HC, healthy control; MMSE, Mini-mental State Examination; MoCA, Montreal Cognitive Assessment; NA, not applicable; PD FOG+, Parkinson's disease with freezing of gait; PD FOG–, Parkinson's disease without freezing of gait; TUG, Timed Up and Go; UPDRS, Unified Parkinson's Disease Rating Scale*.

### WM Abnormalities Identified by TBSS

**Figure 2:**
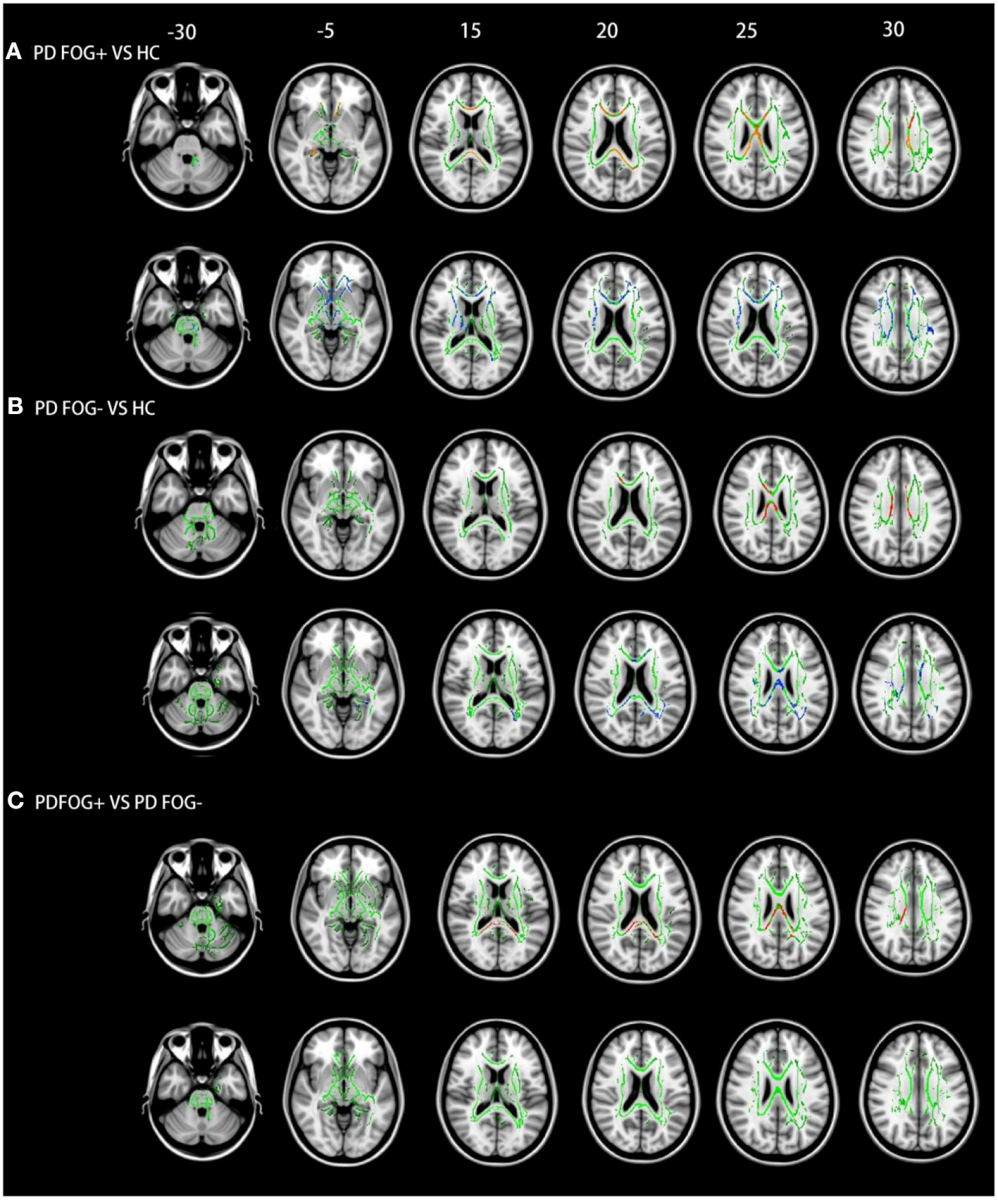
TBSS results in the three groups. **(A)** PD FOG+ vs. HC. **(B)** PD FOG– vs. HC. **(C)** PD FOG+ vs. PD FOG–. Red and blue represent regions with reduced FA and increased MD, respectively. The WM skeleton is shown in green. Results are shown at *P* < 0.05 (family-wise error-corrected).

Compared to HCs, FA in the CC was reduced while MD was increased in the PD FOG+ group (Figure 2A). The same trends were observed in the PD FOG– group—i.e., FA was lower and MD was higher in the CC and corona radiata compared to HCs (Figure 2B). PD FOG+ patients had more WM abnormalities than PD FOG– patients. Notably, FA in the CC was lower in PD FOG+ patients than in PD FOG– patients (Figure 2C), but there was no difference in MD between the two groups.

WM abnormalities were also observed in brain areas outside the CC in PD FOG+ patients: compared to HCs, FA was decreased in the cingulum (hippocampus) and superior longitudinal fasciculus, while MD was increased in the internal capsule, corona radiata, superior longitudinal fasciculus, and thalamus (Figure 2A). In PD FOG– patients, there were no decreases in FA in any WM areas outside the CC; however, increased MD was observed in the cingulum (hippocampus), superior longitudinal fasciculus, thalamus, and external capsule (Figure 2B). Widespread WM changes were observed in the PD FOG+ group compared to the PD FOG– group, including in FA in the cingulum (hippocampus) and superior longitudinal fasciculus and in MD in the internal capsule, corona radiata, superior longitudinal fasciculus, and thalamus (Figure 2C).

### FA and MD in the Splenium, Body, and Genu of the CC

**Figure 3:**
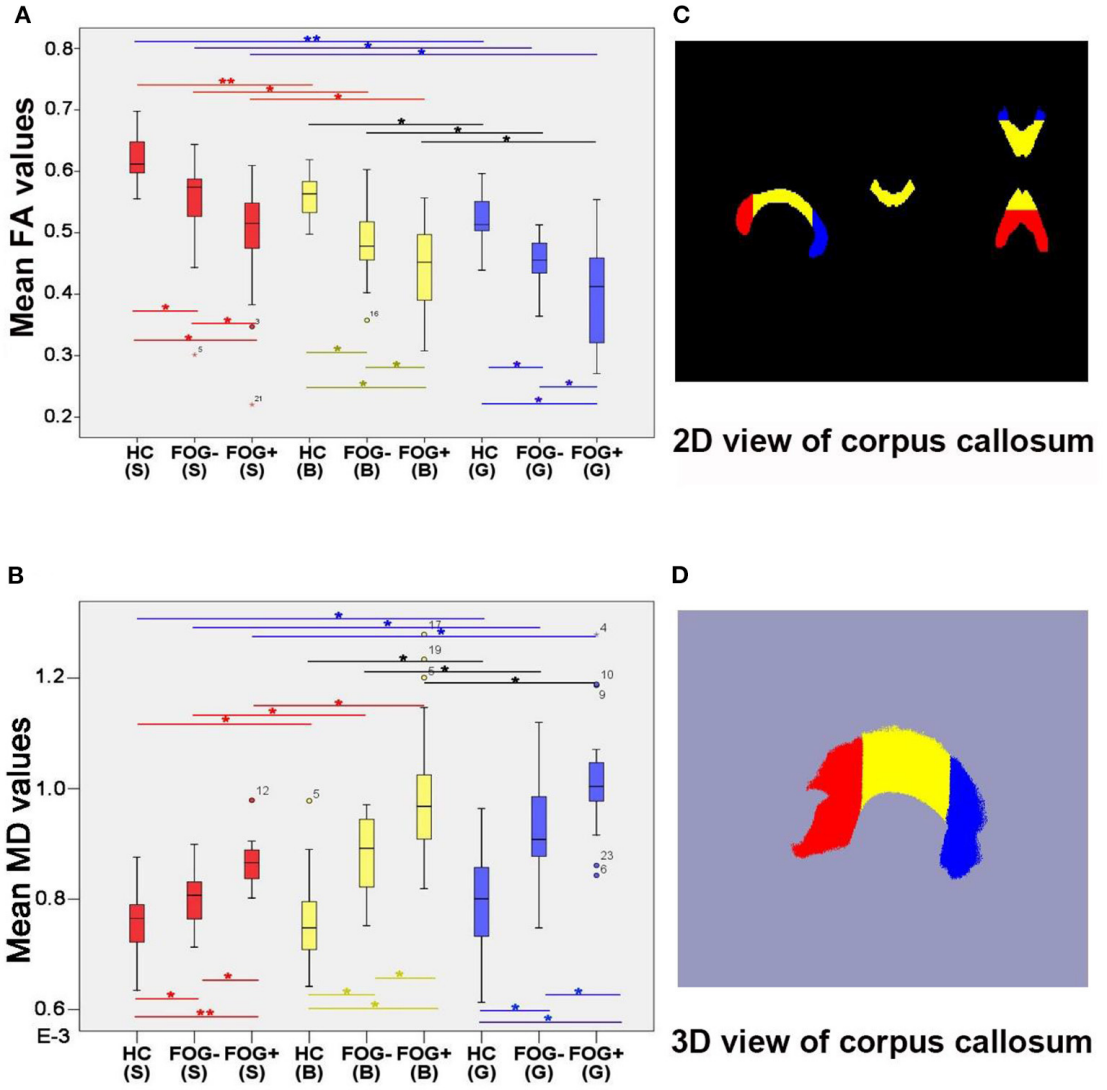
Comparison of FA and MD values of CC splenium, body, and genu. **(A)** Comparison of FA values. **(B)** Comparison of MD values. **(C)** 2D view of CC. **(D)** 3D view of CC. *P < 0.05, **P < 0.01 (two-sample *t*-test; FDR correction was applied to multiple comparisons). B, body; G, genu; S, splenium.

FA and MD differed significantly among the splenium, body, and genu of the CC in the three groups; the rank order of FA values was splenium > body > genu (*P* < 0.05, FDR corrected; Figure 3). PD FOG+ patients had the lowest FA in all three parts of the CC, while PD FOG– patients had lower FA values than HCs (*P* < 0.05, FDR corrected). The median FA values in the splenium were 0.612 for HCs, 0.574 for PD FOG– patients, and 0.515 for PD FOG+ patients; compared to HCs, the values for the PD FOG– and PD FOG+ groups were 6.13 and 15.73% lower, respectively. In the body, the median FA values for PD FOG– patients (0.478) and PD FOG+ patients (0.452) were 15.09 and 19.75% lower, respectively, than that in HCs (0.563). For the genu, the median FA values were 0.455 for PD FOG– patients and 0.412 for PD FOG+ patients, which were 11.30 and 19.67% lower, respectively, than that in HCs (0.513). Thus, the greatest difference in FA between PD patients and HCs was in the body of the CC. The rank order of MD value was body > splenium > genu. The PD FOG+ group had the highest MD in all three parts of the CC, PD FOG– group had lower MD values, and HCs had the lowest values (*P* < 0.05, FDR corrected).

### Correlations Between FA, MD, and FOGQ Score

**Figure 4:**
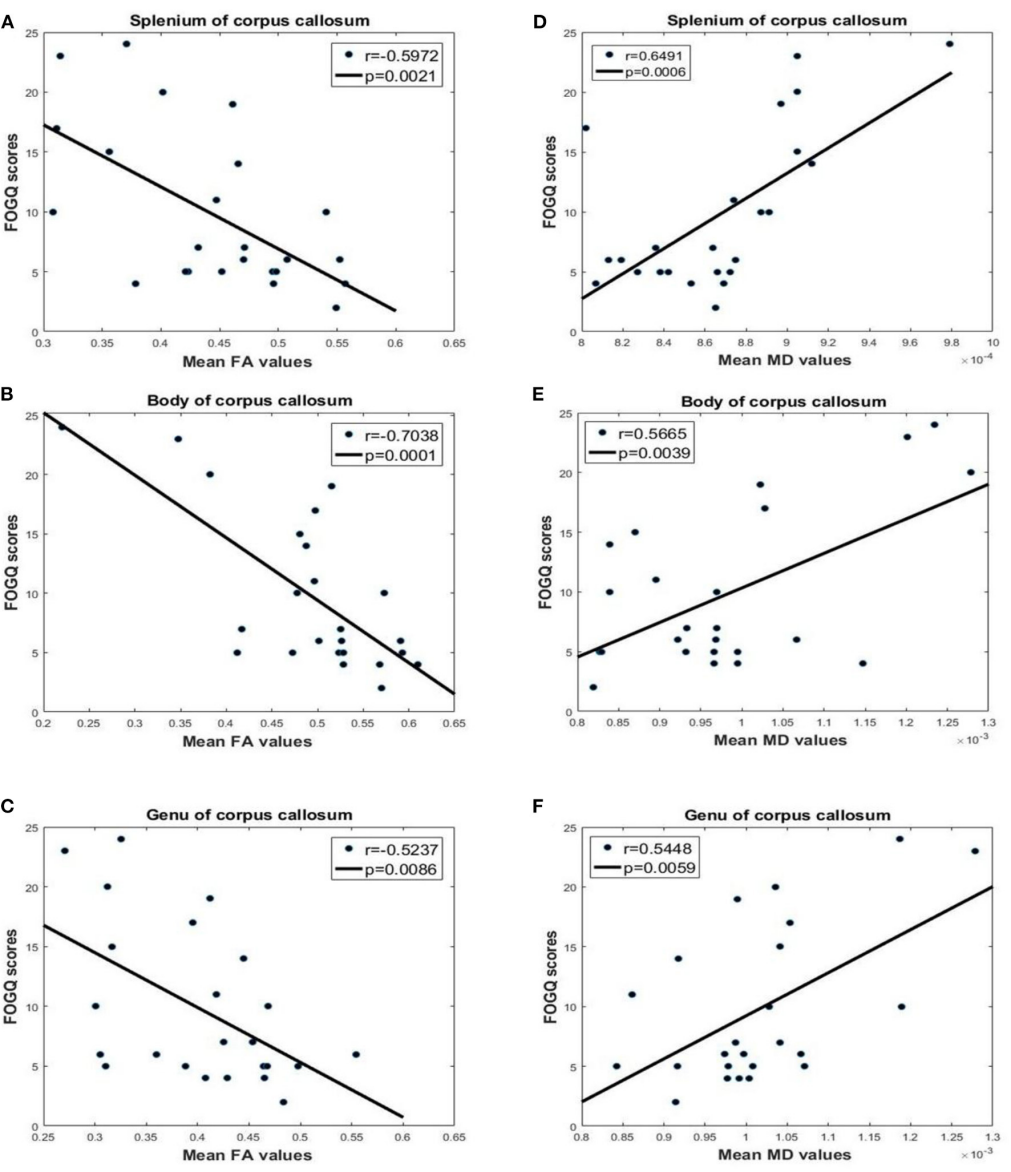
Correlation analysis of FA, MD, and FOGQ scores for the splenium, body, and genu of the CC. FOGQ scores and FA values of the **(A)** splenium, **(B)** body, and **(C)** genu. FOGQ scores and MD values of the **(D)** splenium, **(E)** body, and **(F)** genu.

There was a significantly negative correlation between FA values in the CC and FOGQ scores (splenium: *P* = 0.0021, *r* = −0.5972; body: *P* = 0.0001, *r* = −0.7038; genu: *P* = 0.0086, *r* = −0.5237; Figure 4). This suggests that the integrity of the WM declined with the severity of FOG. MD values in the CC were positively correlated with FOGQ score (splenium: *P* = 0.0006, *r* = 0.6491; body: *P* = 0.0039, *r* = 0.5665; genu: *P* = 0.0059, *r* = 0.5448), indicating that more severe hematogenous edema of the CC was associated with increased FOG severity.

### Correlation Between VMHC and FOGQ Score

**Figure 5:**
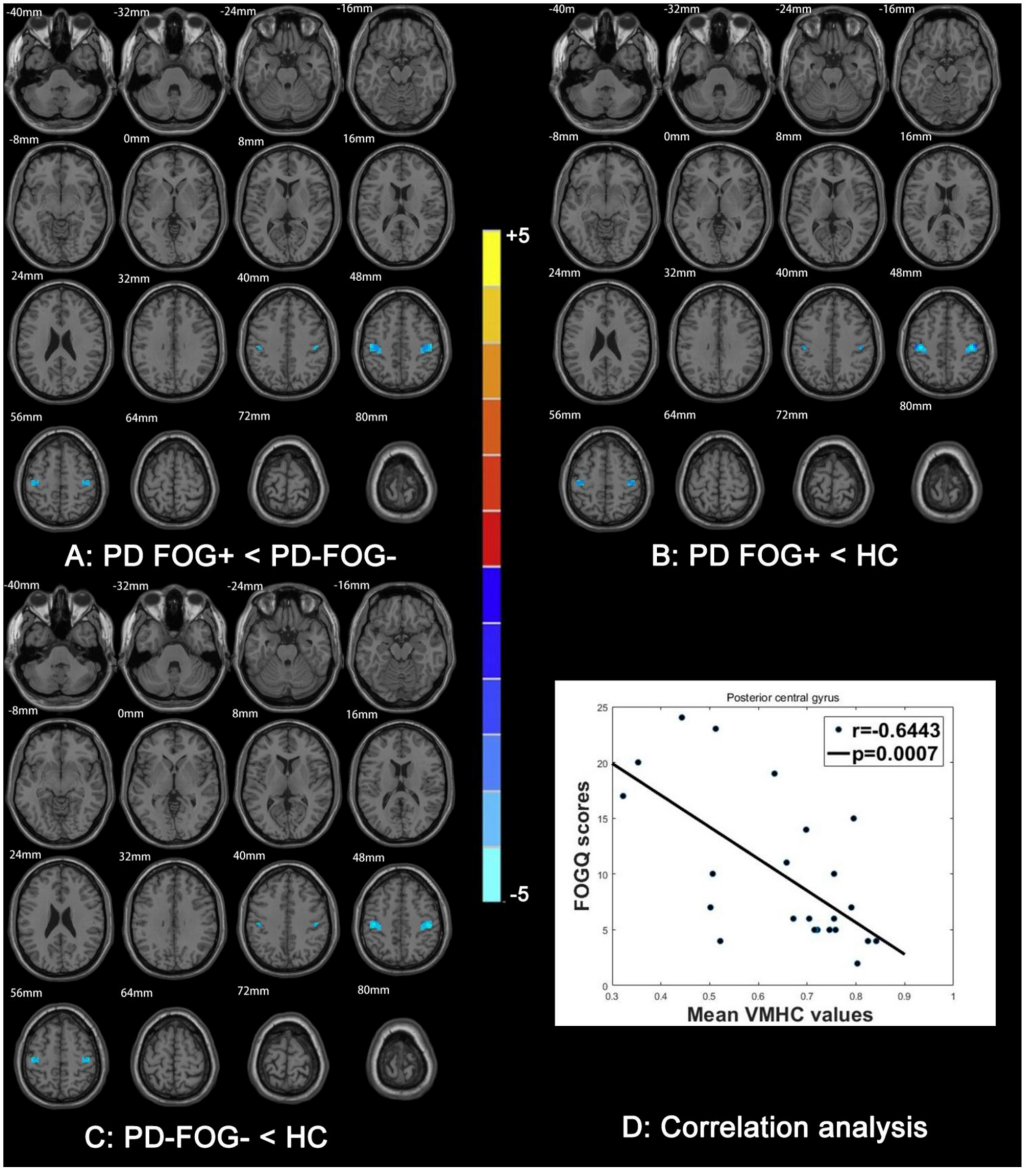
Comparison of VMHC among PD FOG+, PD FOG–, and HC groups. VMHC between **(A)** PD FOG+ and PD FOG–, **(B)** PD FOG+ and HC, and **(C)** PD FOG– and HC. **(D)** Correlation analysis between FOGQ scores and VMHC of PCG.

Compared to HCs, VMHC in the PCG, pre-central gyrus (PRG), and angular gyrus of the parietal inferior margin was reduced in PD FOG+ and PD FOG– patients (Figure 5). Lower VMHC in the PCG, PRG, and parietal inferior margin was observed in PD FOG+ patients compared to PD FOG– patients. In the comparison between PD FOG+ and PD FOG–, the local maxima of the cluster with a significant difference in VMHC was located at *x* = ±48, *y* = −18, and *z* = 48; the *T*-value was −1.351, and the number of voxels in the PCG, PRG, and parietal inferior margin was 130, 51, and 6, respectively (Table 2). The T values for PD FOG+ vs. HC and PD FOG– vs. HC were −5.354 and −4.479, respectively; thus, the difference between PD FOG+ patients and HCs was more significant than that between PD FOG– patients and HCs. FOGQ score was negatively correlated with VMHC in the PCG (*P* = 0.0007, *r* = −0.6443), suggesting that PD patients with more severe FOG have lower PCG functioning.

**Table 2:** VMHC differences among PD FOG+ and PD FOG–patients and HCs.

**Anatomic region (AAL)**	**Number of voxels**	**MNI coordinates of local maxima**			***T*-value**
		***x***	***y***	***z***	
PD FOG+ vs. PD FOG–		±48	−18	48	−1.351
PCG	130				
PRG	51				
Parietal_Inf	6				
PD FOG+ vs. HC		±39	−21	51	−4.479
PCG	118				
PCG	60				
Parietal_Inf	8				
PD FOG– vs. HC		±45	−24	48	−5.354
PCG	120				
PCG	58				
Parietal_Inf	12				

*AAL, Automated Anatomical Labeling; HC, healthy control; Parietal_Inf, parietal inferior margin; PD FOG+, MNI, Montreal Neurological Institute; Parkinson's disease with freezing of gait; PD FOG–, Parkinson's disease without freezing of gait; PCG, post-central gyrus; PRG, pre-central gyrus; VMHC, voxel-mirrored homotopic connectivity*.

### Integration of FA, MD, and VMHC

**Figure 6:**
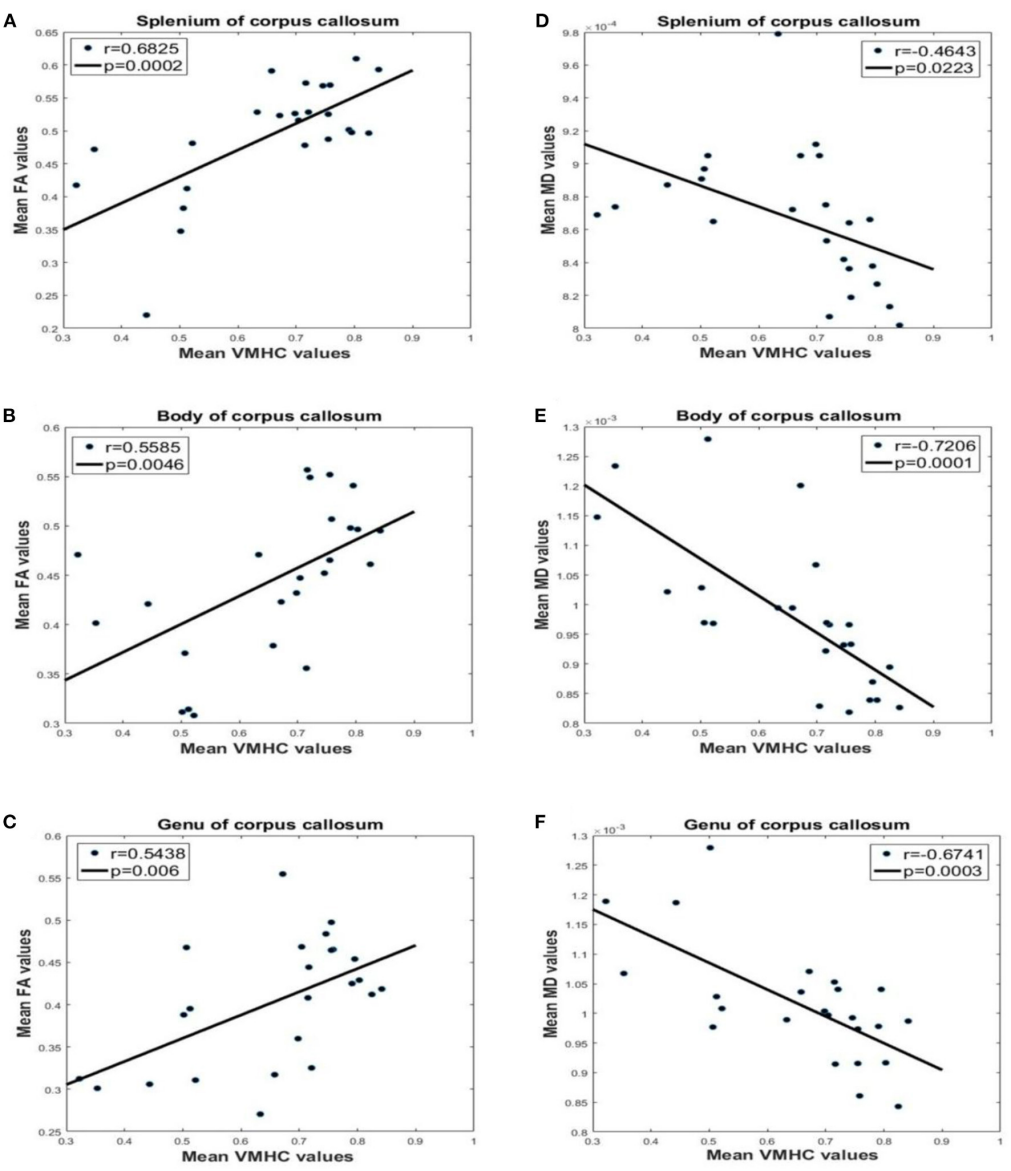
Correlation analysis between FA, MD, and VMHC values for different parts of the CC. VMHC and FA in the **(A)** splenium, **(B)** body, and **(C)** genu. VMHC and MD in the **(D)** splenium, **(E)** body, and **(F)** genu.

FA of the CC was positively correlated with VMHC in the PCG (splenium: *P* = 0.0002, *r* = 0.6825; body: *P* = 0.0046, *r* = 0.5585; genu: *P* = 0.0060, *r* = 0.5438; Figure 6). On the other hand, there was a negative correlation between MD in the CC and VMHC in the PCG (splenium: *P* = 0.0223, *r* = −0.4643; body: *P* = 0.0001, *r* = −0.7206; genu: *P* = 0.0003, *r* = −0.6741). This suggests that the abnormalities in the CC of PD FOG+ patients are related to a decrease in VMHC in the PCG.

## Discussion

The present study investigated changes in brain structure and FC in patients with PD with or without FOG. WM impairment was identified by TBSS, and FA and MD in the CC were compared among groups, and their correlations with FOGQ score were analyzed. VMHC analysis can reveal brain regions with abnormal interhemispheric FC. There were four major findings from our study: (1) structural connectivity was decreased in all three parts of the CC in patients with PD; (2) widespread impairment in the frontal lobe WM was present in PD patients; (3) PD FOG+ patients showed decreased VMHC in the PCG, PRG, and angular gyrus of the parietal inferior margin; and (4) FA, MD, VMHC, and FOGQ score were correlated.

### Decreased Interhemispheric Structural Connectivity in the CC

The TBSS analysis revealed decreased FA and increased MD values in the CC of PD FOG+ patients compared to PD FOG– patients and HCs, indicating that WM microstructure in the CC was abnormal. The CC is the largest connecting tract between the left and right brain hemispheres (28); lesions in the CC result in decreased interhemispheric structural connectivity, which can disrupt brain functions requiring interhemispheric coordination such as cognition, executive functions, and attention. All of these resources are required for gait, especially in unfamiliar environments (25, 29, 30). Thus, our findings suggest that limited cognition/attention/execution resources resulting from CC impairment underlie FOG in PD.

Fiber tracts for leg motor control are located in the CC; a DTI study revealed callosal leg motor fibers passing through the isthmus of the CC (31), which has been confirmed using Klingler's fiber dissection technique (32). Therefore, the impairment in CC (especially splenium) might be associated with FOG in PD patients.

### Widespread Impairment of Frontal Lobe WM

We observed widespread impairment in frontal WM beyond the CC in PD FOG+ patients. This is in line with a previous study in which PD patients with postural imbalance and gait difficulty were found to have a lower FA in the prefrontal cortex than tremor-dominant patients (33). A loss of integrity of WM tracts including superior and inferior longitudinal fasciculus, pedunculopontine nucleus, and corticospinal tract has also been observed in PD patients with FOG (34). Frontal areas are important for higher-order motor control (35); thus, frontal WM disease is another possible cause of PD FOG+ and gait disorder. It should be noted that prefrontal and premotor fibers pass through the anterior part and body of the CC, respectively (31), which are associated with higher-order motor control in the frontal lobe. The lower FAB score in the PD FOG+ group was also indicative of frontal lobe defects.

### Impaired Interhemispheric Functional Coordination in Sensorimotor and Visuospatial Regions

In PD FOG+ patients, VMHC was decreased in the PCG and PRG, which are critical for sensory and motor functions, respectively. This is consistent with several previous fMRI studies reporting a decreased FC between the sensorimotor network and other brain regions (36, 37). A decreased VMHC was also observed in the angular gyrus of the parietal inferior margin in the PD FOG+ group. This is an important component of the frontoparietal network for visuospatial function, and its impairment can lead to left–right disorientation (38, 39). Our results indicate that PD FOG+ patients have visuospatial dysfunction resulting from structural or functional alterations in the frontoparietal network (40).

Gait is neither a sensorimotor skill (e.g., like daily hand motor skill) nor an automatized motor action. A growing body of evidence indicates that gait has the multidimensionality and relies on high cognitive aspects including attention, executive control, and sensorimotor integration, which are all especially impaired in FOG (41–45). It will be interrupted by freezing of gait in PD, representing a pathological condition that can be resolved by a sensory cue. The impaired interhemispheric functional coordination in sensorimotor and visual–spatial regions might be associated with the pathological condition in PD FOG+.

### FA, MD, and VMHC as Potential Biomarkers of FOG Severity

FA and MD in the CC and VMHC in the PCG, PRG, and parietal inferior margin were significantly correlated with the FOGQ score. Other indices such as FC in the sensorimotor network were reported to be correlated with the severity of motor impairment (46). VMHC in the inferior parietal lobe was shown to be negatively correlated with FOGQ score (15). This implies that FA, MD, and VMHC are potential quantitative biomarkers of FOG severity that can be used in conjunction with FOGQ score—which has large interobserver variability—to reduce the risk of misdiagnosing FOG.

### Integrating Structural Connectivity and FC

This is the first study to establish correlations between FA, MD, and VMHC from the same PD FOG+ dataset. FA and MD measure the structural connectivity (i.e., in WM), while VMHC reflects functional homotopic FC. The structural and functional data were concordant and indicated that interhemispheric brain connectivity is impaired in PD FOG+. Thus, integrative analysis of structural connectivity and FC can provide detailed insight into the mechanisms of FOG in PD.

It should be noted that the interhemispheric fibers subserving the three areas with decreased VMHC (PCG, PRG, and parietal inferior margin) likely intersect in the body of the CC. A recent study reported that transection of the CC decreased VMHC, with a greater reduction in the frontal and parietal lobes than in sensorimotor and visual areas (47). The areas of decreased VMHC found in our study were less affected than would be expected following complete and partial callosotomy. Nonetheless, the impairment of the CC in our PD FOG+ patients presumably decreased interhemispheric coordination, leading to visuospatial and sensorimotor dysfunction and loss of the leg motor control.

There were limitations to our study. First, the sample size was relatively small, which may have reduced the statistical power. Second, the asymmetry of cortical structures could affect VMHC, although asymmetric template in fMRI data processing was used to reduce this effect. Third, as our study was cross-sectional, changes in related regions and indices in the progression of FOG– to FOG+ could not be examined. In future work, we will investigate the structural and functional brain networks of PD constructed from multimodal MRI data (34, 48–50) and identify potential network biomarkers of PD FOG+ using machine learning algorithms (51, 52).

## Conclusion

PD FOG+ was found to be associated with abnormal interhemispheric brain connectivity as measured by FA, MD, and VMHC, which were correlated with clinical FOG severity. Our results suggest that decreased FA in the CC impairs all advanced brain functions requiring interhemispheric coordination, and decreased VMHC in three brain regions (PCG, PRG, and parietal inferior margin) underlies defects in visuospatial and sensorimotor functions in FOG. Additionally, our data indicate that FA, MD, and VMHC are potential biomarkers of FOG severity in PD patients and demonstrate that integrating structural and functional MRI measures can provide novel insight into the pathophysiological mechanisms of FOG.

## Data Availability Statement

The original contributions presented in the study are included in the article/supplementary material, further inquiries can be directed to the corresponding author/s.

## Ethics Statement

The studies involving human participants were reviewed and approved by the ethics committee of Guangzhou First People's Hospital. The patients/participants provided their written informed consent to participate in this study.

## Author Contributions

CJ performed experiments and analyzed the data along with SQ. SQ, YY, and XW conceived the study, presented the results, and wrote the manuscript along with CJ. XR collected and analyzed the data. YT and CL supervised the algorithm development and analyzed the data. All authors read and approved the final manuscript.

## Conflict of Interest

The authors declare that the research was conducted in the absence of any commercial or financial relationships that could be construed as a potential conflict of interest.

## Abbreviations

CC: corpus callosum
CSF: cerebrospinal fluid
DTI: diffusion tensor imaging
EPI: echo planar imaging
FA: fractional anisotropy
FAB: Frontal Assessment Battery
FC: functional connectivity
FDR: false discovery rate
fMRI: functional magnetic resonance imaging
FOG: freezing of gait
FOGQ: freezing of gait questionnaire
FOV: field of view
HARS: Hamilton Anxiety Rating Scale
HC: healthy control
HDRS: Hamilton Depression Rating Scale
H&Y: Hoehn and Yahr
MD: mean diffusivity
MMSE: Mini-mental State Examination
MoCA: Montreal Cognitive Assessment
MRI: magnetic resonance imaging
PCG: post-central gyrus
PD: Parkinson's disease
PD FOG+: Parkinson's disease with freezing of gait
PD FOG–: Parkinson's disease without freezing of gait
PRG: pre-central gyrus
TBSS: tract-based spatial statistics
TE: echo time
TR: repetition time
TUG: timed up and go
UPDRS: Unified Parkinson's Disease Resting Scale
VMHC: voxel-mirrored homotopic connectivity
WM: white matter.

## References

[1] Nutt JG Bloem BR Giladi N Hallett M Horak FB Nieuwboer A. Freezing of gait: moving forward on a mysterious clinical phenomenon. Lancet Neurol. (2011) 10:734–44. 10.1016/S1474-4422(11)70143-0 21777828 PMC7293393

[2] Wang M Jiang S Yuan Y Zhang L Ding J Wang J. Alterations of functional and structural connectivity of freezing of gait in Parkinson's disease. J Neurol. (2016) 263:1583–92. 10.1007/s00415-016-8174-4 27230857

[3] Bensimon G Ludolph A Agid Y Vidailhet M Payan C Leigh PN. Riluzole treatment, survival and diagnostic criteria in Parkinson plus disorders: the NNIPPS study. Brain. (2009) 132:156–71. 10.1093/brain/awn291 19029129 PMC2638696

[4] Shine JM Naismith SL Lewis SJ. The pathophysiological mechanisms underlying freezing of gait in Parkinson's Disease. J Clin Neurosci. (2011) 18:1154–7. 10.1016/j.jocn.2011.02.007 21724402

[5] Litvan I Agid Y Calne D Campbell G Dubois B Duvoisin RC. Clinical research criteria for the diagnosis of progressive supranuclear palsy (Steele-Richardson-Olszewski syndrome): report of the NINDS-SPSP International Workshop. Neurology. (1996) 47:1–9. 10.1212/wnl.47.1.1 8710059

[6] Nieuwboer A Giladi N. Characterizing freezing of gait in Parkinson's disease: models of an episodic phenomenon. Mov Disord. (2013) 28:1509–19. 10.1002/mds.25683 24132839

[7] Bächlin M Plotnik M Roggen D Maidan I Hausdorff JM Giladi N. Wearable assistant for Parkinson's disease patients with the freezing of gait symptom. IEEE Trans Inf Technol Biomed. (2010) 14:436–46. 10.1109/TITB.2009.2036165 19906597

[8] Youn J Cho JW Lee WY Kim GM Kim ST Kim HT. Diffusion tensor imaging of freezing of gait in patients with white matter changes. Mov Disord. (2012) 277:760–4. 10.1002/mds.24034 22162037

[9] Fling BW Cohen RG Mancini M Carpenter SD Fair DA Nutt JG. Functional reorganization of the locomotor network in Parkinson patients with freezing of gait. PLoS ONE. (2014) 9:e100291. 10.1371/journal.pone.0100291 24937008 PMC4061081

[10] Pietracupa S Suppa A Upadhyay N Giannì C Grillea G Leodori G. Freezing of gait in Parkinson's disease: gray and white matter abnormalities. J Neurol. (2018) 26:552–62. 10.1007/s00415-017-8654-1 29128929

[11] Stark DE Margulies DS Shehzad ZE Reiss P Kelly AM Uddin LQ. Regional variation in interhemispheric coordination of intrinsic hemodynamic fluctuations. J Neurosci. (2008) 28:13754. 10.1523/JNEUROSCI.4544-08.2008 19091966 PMC4113425

[12] Fox MD Raichle ME. Spontaneous fluctuations in brain activity observed with functional magnetic resonance imaging. Nat Rev Neurosci. (2007) 8:700–11. 10.1038/nrn2201 17704812

[13] Zuo XN Kelly C Di Martino A Mennes M Margulies DS Bangaru S. Growing together and growing apart: regional and sex differences in the lifespan developmental trajectories of functional homotopy. J Neurosci. (2010) 30:15034–43. 10.1523/JNEUROSCI.2612-10.2010 21068309 PMC2997358

[14] Luo C Guo X Song W Zhao B Cao B Yang J. Decreased resting-state interhemispheric functional connectivity in Parkinson's disease. Biomed Res Int. (2015) 2015:692684. 10.1155/2015/692684 26180807 PMC4477209

[15] Li J Yuan Y Wang M Zhang J Zhang L Jiang S. Decreased interhemispheric homotopic connectivity in Parkinson's disease patients with freezing of gait: a resting state fMRI study. Parkinsonism Relat Disord. (2018) 52:30–6. 10.1016/j.parkreldis.2018.03.015 29602542

[16] Canu E Agosta F Sarasso E Volontè MA Basaia S Stojkovic T. Brain structural and functional connectivity in Parkinson's disease with freezing of gait. Hum Brain Mapp. (2015) 36:5064–78. 10.1002/hbm.22994 26359798 PMC6869160

[17] Chung SJ Lee YH Yoo HS Oh JS Kim JS Ye BS. White matter hyperintensities as a predictor of freezing of gait in Parkinson's disease. Parkinsonism Relat Disord. (2019) 66:105–9. 10.1016/j.parkreldis.2019.07.019 31324555

[18] Giladi N Shabtai H Simon ES Biran S Tal J Korczyn AD. Construction of freezing of gait questionnaire for patients with Parkinsonism. Parkinsonism Relat Disord. (2000) 6:165–70. 10.1016/s1353-8020(99)00062-0 10817956

[19] Movement Disorder Society Task Force on Rating Scales for Parkinson's Disease. The Unified Parkinson's Disease Rating Scale (UPDRS): status and recommendations. Mov Disord. (2003) 18:738–50. 10.1002/mds.10473 12815652

[20] Diedrichsen J Maderwald S Küper M Thürling M Rabe K Gizewski ER. Imaging the deep cerebellar nuclei: a probabilistic atlas and normalization procedure. NeuroImage. (2011) 54:1786–94. 10.1016/j.neuroimage.2010.10.035 20965257

[21] Kleiner A Pacifici I Vagnini A Camerota F Celletti C Stocchi F. Timed Up and Go evaluation with wearable devices: validation in Parkinson's disease. J Bodyw Mov Ther. (2018) 22:390–5. 10.1016/j.jbmt.2017.07.006 29861240

[22] Datta AK Das D Bhattacharyya KB Bose P Mishra AK Das SK. Frontal assessment battery in Parkinson's disease: a study on 170 patients. Neurol India. (2019) 67:433–8. 10.4103/0028-3886.258052 31085855

[23] Smith SM Jenkinson M Johansen-Berg H Rueckert D Nichols TE Mackay CE. Tract-based spatial statistics: voxelwise analysis of multi-subject diffusion data. NeuroImage. (2006) 31:1487–505. 10.1016/j.neuroimage.2006.02.024 16624579

[24] Chao-Gan Y Yu-Feng Z. DPARSF: a MATLAB toolbox for “pipeline” data analysis of resting-state fMRI. Front Syst Neurosci. (2010) 4:13. 10.3389/fnsys.2010.00013 20577591 PMC2889691

[25] Bharti K Suppa A Tommasin S Zampogna A Pietracupa S Berardelli A. Neuroimaging advances in Parkinson's disease with freezing of gait: a systematic review. Neuroimage Clin. (2019) 24:102059. 10.1016/j.nicl.2019.102059 31795038 PMC6864177

[26] Jia X-Z Wang J Sun H-Y Zhang H Liao W Wang Z. RESTplus: an improved toolkit for resting-state functional magnetic resonance imaging data processing. Sci Bull. (2019) 64:953–4. 10.1016/j.scib.2019.05.008 36659803

[27] Fan H Yang X Zhang J Chen Y Li T Ma X. Analysis of voxel-mirrored homotopic connectivity in medication-free, current major depressive disorder. J Affect Disord. (2018) 240:171–6. 10.1016/j.jad.2018.07.037 30075387

[28] Xue C Shi L Hui S Wang D Lam TP Ip CB. Altered white matter microstructure in the corpus callosum and its cerebral interhemispheric tracts in adolescent idiopathic scoliosis: diffusion tensor imaging analysis. Am J Neuroradiol. (2018) 39:1177–84. 10.3174/ajnr.A5634 29674416 PMC7410631

[29] Hall JM Ehgoetz Martens KA Walton CC O'Callaghan C Keller PE Lewis SJ. Diffusion alterations associated with Parkinson's disease symptomatology: a review of the literature. Parkinsonism Relat Disord. (2016) 33:12–26. 10.1016/j.parkreldis.2016.09.026 27765426

[30] Hall JM Shine JM Ehgoetz Martens KA Gilat M Broadhouse KM Szeto J. Alterations in white matter network topology contribute to freezing of gait in Parkinson's disease. J Neurol. (2018) 265:1353–64. 10.1007/s00415-018-8846-3 29616302

[31] Wahl M Lauterbach-Soon B Hattingen E Jung P Singer O Volz S. Human motor corpuscallosum: topography, somatotopy, and link between microstructure and function. J Neurosci. (2007) 27:12132–8. 10.1523/JNEUROSCI.2320-07.2007 17989279 PMC6673264

[32] Naets W Van Loon J Paglioli E Van Paesschen W Palmini A Theys T. Callosotopy: leg motor connections illustrated by fiber dissection. Brain Struct Funct. (2017) 222:661–7. 10.1007/s00429-015-1167-8 26666531

[33] Lenfeldt N Holmlund H Larsson A Birgander R Forsgren L. Frontal white matter injuries predestine gait difficulties in Parkinson'sdisease. Acta Neurol Scand. (2016) 134:210–8. 10.1111/ane.12532 27465659

[34] Allali G Blumen HM Devanne H Pirondini E Delval A Van De Ville D. Brain imaging of locomotion in neurological conditions. Neurophysiol Clin. (2018) 48:337–59. 10.1016/j.neucli.2018.10.004 30487063 PMC6563601

[35] Bohnen NI Jahn K. Imaging: What can it tell us about parkinsonian gait? Mov Disord. (2013) 28:1492–500. 10.1002/mds.25534 24132837 PMC3801220

[36] Lenka A Naduthota RM Jha M Panda R Prajapati A Jhunjhunwala K. Freezing of gait in Parkinson's disease is associated with altered functional brain connectivity. Parkinsonism Relat Disord. (2016) 24:100–6. 10.1016/j.parkreldis.2015.12.016 26776567

[37] Myers PS McNeely ME Pickett KA Duncan RP Earhart GM. Effects of exercise on gait and motor imagery in people with Parkinson disease and freezing of gait. Parkinsonism Relat Disord. (2018) 53:89–95. 10.1016/j.parkreldis.2018.05.006 29754837 PMC6120800

[38] Barthel C Nonnekes J van Helvert M Haan R Janssen A Delval A. The laser shoes: a new ambulatory device to alleviate freezing of gait in Parkinson disease. Neurology. (2018) 90:e164–71. 10.1212/WNL.0000000000004795 29263221

[39] Seghier ML. The angular gyrus: multiple functions and multiple subdivisions. Neuroscientist. (2013) 19:43–61. 10.1177/1073858412440596 22547530 PMC4107834

[40] Bharti K Suppa A Pietracupa S Upadhyay N Giannì C Leodori G. Abnormal cerebellar connectivity patterns in patients with Parkinson's disease and freezing of gait. Cerebellum. (2019) 18:298–308. 10.1007/s12311-018-0988-4 30392037

[41] Verghese A. Physicians and addiction. N Engl J Med. (2002) 346:1510–1. 10.1056/NEJM200205163462002 12015389

[42] Lewis SJ Shine JM. The next step: a common neural mechanism for freezing of gait. Neuroscientist. (2014) 22:72–82. 10.1177/1073858414559101 25398230

[43] Gilat M Martens KAE Miranda-Domínguez O Arpan I Shine JM Mancini M. Dysfunctional limbic circuitry underlying freezing of gait in Parkinson's disease. Neuroscience. (2018) 374:119–32. 10.1016/j.neuroscience.2018.01.044 29408498 PMC6390849

[44] Maidan I Jacob Y Giladi N Hausdorff JM Mirelman A. Altered organization of the dorsal attention network is associated with freezing of gait in Parkinson's disease. Parkinsonism Relat Disord. (2019) 63:77–82. 10.1016/j.parkreldis.2019.02.036 30827838

[45] Li KZH Bherer L Mirelman A Maidan I Hausdorff JM. Cognitive involvement in balance, gait and dual-tasking in aging: a focused review from a neuroscience of aging perspective. Front Neurol. (2018) 9:913. 10.3389/fneur.2018.00913 30425679 PMC6219267

[46] Yoo HS Bak Y Chung SJ Lee Y Ye BS Sohn YH. Impaired functional connectivity of sensorimotor network predicts recovery in drug-induced parkinsonism. Parkinsonism Relat Disord. (2020) 74:16–21. 10.1016/j.parkreldis.2020.03.031 32283491

[47] Roland JL Snyder AZ Hacker CD Mitra A Shimony JS Limbrick DD. On the role of the corpus callosum in interhemispheric functional connectivity in humans. Proc Natl Acad Sci USA. (2017) 114:13278–83. 10.1073/pnas.1707050114 29183973 PMC5740665

[48] Qi S Meesters S Nicolay K Romeny BM Ossenblok P. The influence of construction methodology on structural brain network measures: a review. J Neurosci Methods. (2015) 253:170–82. 10.1016/j.jneumeth.2015.06.016 26129743

[49] Qi S Meesters S Nicolay K Romeny BM Ossenblok P. Structural brain network: what is the effect of LiFE optimization of whole brain tractography? Front Comput Neurosci. (2016) 10:12. 10.3389/fncom.2016.00012 26909034 PMC4754446

[50] Qi S Gao Q Shen J Teng Y Xie X Sun Y. Multiple frequency bands analysis of large scale intrinsic brain networks and its application in schizotypal personality disorder. Front Comput Neurosci. (2018) 12:64. 10.3389/fncom.2018.00064 30123120 PMC6085977

[51] Qian H Qin D Qi S Teng Y Li C Yao Y. Less is better: Single-digit brain functional connections predict T2DM and T2DM-induced cognitive impairment. Front Neurosci. (2021) 14:588684. 10.3389/fnins.2020.588684 33505236 PMC7829678

[52] Zhu Y Qi S Zhang B He D Teng Y Hu J. Connectome-based biomarkers predict subclinical depression and identify abnormal brain connections with the lateral habenula and thalamus. Front Psychiatry. (2019) 10:371. 10.3389/fpsyt.2019.00371 31244688 PMC6581735

